# Telling a good story: The effects of memory retrieval and context processing on eyewitness suggestibility

**DOI:** 10.1371/journal.pone.0212592

**Published:** 2019-02-21

**Authors:** Jessica A. LaPaglia, Jason C. K. Chan

**Affiliations:** 1 Department of Social Sciences, Morningside College, Sioux City, Iowa, United States of America; 2 Department Psychology, Iowa State University, Ames, Iowa, United States of America; University of Westminster, UNITED KINGDOM

## Abstract

Witnesses are likely to describe a crime many times before testifying or encountering misinformation about that crime. Research examining the effect of retrieval on later suggestibility has yielded mixed results. LaPaglia and Chan manipulated whether misinformation was presented in a narrative or misleading questions, and they found that retrieval increased suggestibility when misinformation was presented in a narrative, but reduced suggestibility when the same misinformation was presented in questions. In the current study, we aimed to address why these differences occurred. Specifically, we examined whether contextual detail and narrative coherence during misinformation exposure influenced the relation between retrieval and suggestibility. Participants watched a robbery video and some were questioned about the event afterwards. They were then exposed to misinformation presented in a narrative (Experiment 1) or questions (Experiment 2) before taking a final memory test. Testing enhanced suggestibility when the misinformation phase reinstated contextual information of the event, but not when the misinformation phase included few contextual details–regardless of whether the misinformation was in a narrative or questions. In Experiment 3, disrupting narrative coherence by randomizing the order of contextual information eliminated retrieval-enhanced suggestibility. Therefore, context processing during the post-event information phase influences whether retrieval enhances or reduces eyewitness suggestibility.

## Introduction

The process of criminal proceedings is slow. For example, it took the city of New York an average of over 600 days to bring a trial before a jury in 2016 [1]. As a result, a witness may have to wait months or even years before providing testimony in court, during which there are many opportunities for a witness to encounter misinformation and recall the witnessed event to people such as a 911 operator, police officers, attorneys, friends, family, and colleagues. Decades of research have shown the damaging effects of misinformation on the accuracy of eyewitness testimony (i.e., the misinformation effect) [2]. Critically, recent research has shown that recalling an event can increase eyewitness suggestibility to misinformation that is presented later—a finding known as Retrieval-Enhanced Suggestibility (RES) [3,4]. These studies often follow the four-phase paradigm introduced by Chan, Thomas, and Bulevich [5]. First, participants watch a video of the witnessed event. Second, some participants receive an initial test over several details presented in the video (simulating a police interview) whereas others do not. Third, all participants are provided with a narrative that contains misinformation about the witnessed event. Lastly, all participants receive a final test for the video (simulating providing testimony during deposition or in trial). The canonical but unexpected result from this paradigm is that attempting to recall witnessed event details before being exposed to misinformation increases the negative influence of that misinformation [6]. This RES effect is surprising because retrieving memories in a memory test typically enhances memory retention (i.e., the retrieval practice effect, a.k.a. the testing effect) [7] and should protect eyewitnesses against later misinformation. Indeed, several studies have demonstrated that prior memory testing can reduce eyewitness suggestibility [8–11]. Therefore, an important question is what factors drive these different effects of retrieval.

LaPaglia and Chan [12] examined whether variations in misinformation presentation format (narrative vs. questions) affected RES in two experiments. They showed participants a video that depicted a bank robbery and a hostage situation. After half of the participants had taken an initial memory test, they were exposed to misinformation via either an audio narrative (Experiment 1) or a set of questions (Experiment 2). When misinformation was presented in a narrative, participants listened to a synopsis of the events that occurred in the bank robbery, and the narration proceeded at a natural pace. But embedded in some sentences were misleading details. For example, the narrative began with “the scene opens with the front of City Towers Bank during the day. Inside, the manager, Ms. Ruth Skellar, is meeting with employees discussing the bank’s ranking in the area.” In this example, the misinformation was “City Towers Bank,” because the actual name of the bank was “City Central Bank.” In this condition, participants simply needed to listen to the narrative carefully. In contrast, when misinformation was presented via questions (in their Experiment 2), participants were shown individual questions on a computer screen for which they provided a response. The question equivalent of the previous narrative example was “In the first scene of City Towers Bank, what is the manager, Ruth Skellar, discussing with her employees?” Embedded in some questions was a piece of misinformation that contradicted a critical detail (e.g., the name of the bank), and participants were asked to recall a noncritical detail (e.g., what Ruth discussed with the employees). Later, participants’ memory for the video was tested again on a final test. LaPaglia and Chan found that taking a memory test immediately after the watching the witnessed event video may participants more susceptible to misinformation when it was presented in a narrative—an RES effect. However, they found that prior retrieval *reduced* suggestibility (i.e., a testing effect) when misinformation was embedded in questions. Although a few prior studies have examined the impact of different misinformation presentation formats (i.e., questions vs. narrative) on eyewitness suggestibility—without the inclusion of an initial memory test—the effects are typically small [13,14]. LaPaglia and Chan’s [12] study was the first to show that prior retrieval can produce opposite effects on eyewitness suggestibility depending on how misinformation is presented. Below, we describe a theoretical account of this intriguing reversal; we then test this account in three experiments.

Before addressing why prior retrieval can increase or decrease suggestibility depending on how misinformation is presented, we first introduce concepts that help explain why RES occurs in the first place. Here, the most prominent explanation suggests that prior testing increases suggestibility because it enhances later learning of the misinformation—that is, prior testing potentiates learning of the misinformation [3,5,15–18]. For exposition purposes, we term this the test-potentiated learning (TPL) account. According to this explanation, when witnesses are asked specific questions about a witnessed event, these questions serve to emphasize the importance of these event details. When witnesses encounter new, or misleading, information relevant to these questions later, their attention is drawn toward this new information, thus improving its encoding and increasing its likelihood of being reported later. For example, a witness may be asked to recall the type of vehicle driven by a perpetrator. Later, the witness’s attention may be inadvertently drawn to the postevent information when it contains information about the type of vehicle, thereby enhancing its encoding.

The above example refers to a *specific* mechanism by which prior testing can potentiate learning of later misinformation (i.e., it enhances encoding of items for which people were queried about previously). There is, however, an additional, more *general* mechanism by which testing can enhance misinformation encoding. A substantial literature has shown that performing retrieval practice can strengthen learning of subsequent material, even if that subsequent material is unrelated to the tested, previously learned material [19–21]. This more general TPL effect has been ascribed to the ability for retrieval to reduce proactive interference from prior learning [22], reduce mind wandering [23], promote a mental context shift [24,25], and optimize one’s encoding strategies [6,26,27]. For present purposes, regardless of the exact mechanisms (i.e., specific or general) by which testing potentiates subsequent learning, the TPL account places a heavy emphasis on the enhanced *encoding of misinformation* as the key contributor to RES. Consequently, factors that affect the encoding of misinformation should impact how retrieval affects suggestibility. To this end, we contend that retrieval increases suggestibility for narrative-based misinformation but decreases suggestibility for question-based misinformation because these presentation methods elicit different encoding processes from participants. In the following, we invoke the ideas of fuzzy-trace theory [28] to specify how this occurs.

According to fuzzy-trace theory, when people encode information (e.g., they see the City Central Bank appear on the witnessed event video), a verbatim memory trace and a gist memory trace are generated in parallel, with the former containing specific information (e.g., City Central Bank) and the latter containing more general, gist-based information (e.g., bank) [29,30]. In the misinformation paradigm, when participants encounter erroneous information (e.g., City Tower Bank), they generate another verbatim trace (e.g., City Tower Bank) that interferes with the original verbatim trace and a gist trace (bank) that further enhances the original gist trace [31]. False memories are recalled when the witness fails to access the verbatim trace of the original event and instead accesses the gist trace of the original event and/or the verbatim trace of the misinformation. Applying these ideas to the present context, initial testing is expected to increase the narrative-based misinformation effect because it potentiates learning of the misinformation by enhancing both verbatim and gist processing during the misinformation exposure phase. Stronger verbatim processing of the misinformation is expected to increase its interfere with retrieval of the original verbatim trace, and stronger gist processing during the misinformation exposure phase may promote integration of the misinformation into the contextual representation of the overall witnessed event. Together, these processes are expected to produce an RES effect.

The more intriguing question is why initial testing would weaken the question-based misinformation effect (that is, eliminating or reversing RES)? Here, we argue that presenting misinformation in questions may have eliminated the RES effect because the question presentation format biased encoding operations away from gist processing and towards verbatim processing. Specifically, in LaPaglia and Chan [12], the questions were presented individually without being accompanied by the contextual information that generates the story-like structure of the narrative presentation format. Table 1 provides an example of our operational definitions for contextual and critical details. Participants in the question condition had far fewer contextual details reinstated from the original witnessed event, which should *reduce gist processing and encourage verbatim processing*. Here, we hypothesize that this bias towards verbatim processing allows prior testing to reduce the influence of the misinformation via two mechanisms. First, the weakened gist processing should reduce the likelihood that the misinformation would be integrated into the contextual representation of the original witnessed event. Second and more importantly, the bias toward verbatim processing may encourage participants to retrieve the verbatim trace of the witnessed event during the misinformation exposure phase. Because prior testing strengthens the verbatim trace of the original memory, the tested participants should be particularly likely to spontaneously retrieve this prior verbatim trace, thereby enhancing their ability to detect discrepancies between their memory and the misinformation, thus allowing them to reject the latter [32,33].

**Table 1:** An example of a narrative that contains contextual details compared to their corresponding misleading questions without contextual details.

Narrative Presentation	Question Presentation
The scene opens with the front of **City Towers Bank** during the day. Inside, the manager, Ms. Ruth Skellar, is meeting with employees discussing the bank’s ranking in the area. She explains that the bank’s performance has vastly improved over the last 12 months and congratulates Wendy Trailer for having the top sales in their division. During the meeting, an employee named Paul walks in late and begins to apologize. Once Ruth dismisses everyone, she asks to have a word with Paul. He attempts to apologize again but Ruth cuts him off asking him to straighten out his priorities. Paul leaves and Ruth asks that someone at the front desk to call the alarm company because **the alarm had mistakenly gone off** earlier in the day. She proceeds into her office when the door is shut behind her. A robber dressed in all black and a ski mask points a gun at her, hands her two bags, and asks for $500,000 in cash.	In the first scene in **City Towers Bank**, what is the manager, Ruth Skellar, discussing with her employees? Who did Ruth ask to call the security company about **the alarm mistakenly going off**?

Misleading details are displayed in bold text; contextual details are underlined. Note that bolding and underlining are used purely to isolate the critical details. In the actual experiments, participants never see any bolded or underlined text.

According to this Context Processing Account, it is differences in contextual information (i.e., rich vs. impoverished context), rather a difference in the specific presentation format (i.e., narrative vs. question) that produced the opposite effects of suggestibility observed in LaPaglia and Chan’s study [12]. Although this account was generated based on how LaPaglia and Chan implemented their narrative vs. question presentation, our results can inform the mechanisms regarding eyewitness suggestibility beyond the confines of the LaPaglia and Chan study. From an eyewitness memory perspective, the present study highlights an important component of the misinformation effect that has traditionally been overlooked [13,34]–the condition under which people encounter misinformation may have a powerful impact on their suggestibility. Moreover, the aforementioned account, as will be evident later, furthers our understanding on the complex interplay between retrieval and subsequent learning of new information—a topic of research that is gaining increasing interest [19–21,35,36].

Three experiments were designed to test the Context Processing Account described above. We adopted the materials used by LaPaglia and Chan [10], and presented the post-event information visually to all participants. That is, all participants read the misinformation narrative or answered the misinformation questions on a computer screen. In Experiment 1, participants were exposed to misinformation through a narrative, but we manipulated whether the narrative included contextual details (i.e., *with-context condition*) or not (*without-context condition*). In Experiment 2, participants were exposed to misinformation through questions, and we again manipulated the inclusion/exclusion of contextual details. In Experiment 3, the misinformation was presented in a narrative, but instead of manipulating the inclusion/exclusion of contextual details, we attempted to directly manipulate gist processing by varying the presentation order of the contextual details. In the *coherent presentation condition*, sentences that contained contextual details were presented in a coherent, sequential order. However, in the *random presentation condition*, we shuffled presentation order of the contextual sentences. Randomizing the contextual sentence presentation order served to increase comprehension difficulty of the narrative [37–40] and decrease gist processing without reducing the amount of contextual details presented. Note that presentation order of the critical sentences were held constant across the coherent and random presentation conditions, so that the misinformation was presented at the same location for all participants.

Across all three experiments, we predicted that an RES effect would occur when the misinformation exposure phase promotes gist processing by reinstating the original context of the witnessed event (i.e., the with-context condition in Experiments 1 and 2 and the coherent presentation condition in Experiment 3). In contrast, the RES effect should not occur when the misinformation exposure phase discourages gist processing, such as when contextual processing is made difficult (i.e., the without-context condition in Experiments 1 and 2 and the random presentation condition in Experiment 3). Before turning to the individual experiments, we emphasize here that the Context Processing Account does not possess the level of theoretical specificity that allows us to predict whether a particular manipulation would *reverse* the RES effect. Instead, they provide the reasoning for when RES is more and less likely to occur. Consequently, we have described the above predictions in terms of an RES effect and its elimination. In reality, the influence of prior retrieval on suggestibility can range from negative (i.e., RES) to positive (i.e., testing effect).

## Experiment 1

### Method

#### Participants and design

Experiment 1 used a 2 (initial test condition: tested vs. control) X 2 (misinformation format: with context vs. without context) X 2 (postevent information: neutral vs. misled) mixed design. Initial test condition and contextual detail were manipulated between subjects. Postevent information was manipulated within subjects. Participants were 120 undergraduate students from a large Midwestern university who participated for partial course credit (71 female; mean age = 18.77, *SD* = 1.16). Thirty participants were included in each between-subjects condition. All experiments reported herein were approved by the Iowa State University Institutional Review Board under the approval number 08–256. All participants signed a written informed consent prior to participation.

### Materials and procedure

#### Video stimulus

Participants began by watching a 25 min video from an episode of the television show *Flashpoint*, which was the same video used by LaPaglia and Chan [12]. The video depicted a disgruntled former employee robbing a bank. Participants were given intentional encoding instructions. They were told to pay close attention to the video, including the actions and surrounding environment, because their memory would be tested later.

#### Initial test and distractor phase

Following the video, the control participants played the video game Tetris for 7 min as a distractor activity, whereas the tested participants took a cued recall test over their memory for the video. This test consisted of 14 open-ended questions; none of which included any misleading information (e.g., “How many warning shots did the robber fire?”). Participants were given 30 s to answer each question, and the initial test phase lasted 7 min. They were told to be as accurate as possible and not to guess. No corrective feedback was given. Once participants completed the initial test or Tetris, they were shown another video to fill a 20 min retention interval. The video was from the BBC show *Spooks* and depicted the British Security Service attempting to foil a terrorist plot. Participants were told that they should pay close attention to the video, but they were not told whether their memory for the video would be tested later.

#### Misinformation narrative

Following the distractor video, participants were presented with a narrative that contained misinformation, with sentences presented in blocks *visually on a computer screen* (see S1 Appendix). Each block contained one to five sentences and was presented on its own screen. All 14 critical details that were questioned in the initial test were included in the narrative. The critical details were either presented as misinformation or a neutral detail. For example, one critical detail was the number of warning shots the robber fired. If this detail was presented as misinformation, the participants read that the robber fired *two* warning shots when he had in fact fired three. For example, the sentence read, “Inside the bank, Ruth expresses concern for a woman with asthma who needs her medication. This angers the robber so he grabs Ruth pointing a gun at her once again. He fires *two warning shots* into the ceiling.” If this detail was presented as a neutral item, participants read, “Inside the bank, Ruth expresses concern for a woman with asthma who needs her medication. This angers the robber so he grabs Ruth pointing a gun at her once again. He fires *warning shots* into the ceiling.” These sentences are identical except that the number of shots fired was not specified in the neutral condition. Across the 14 critical details, seven were presented as a misled item and seven were presented as a neutral item. Whether a given detail was presented as a misled or neutral detail was counterbalanced across participants.

Participants in the with-context condition read the entire narrative, which presented 28 blocks of information (i.e., 14 blocks of critical information and 14 blocks of contextual information). In the without-context condition, only the 14 blocks that contained the critical details were shown to participants. All participants were given 30 s to read each block of text. Most importantly, presentation duration of the critical details was held constant for all participants (i.e., all were given 30 s to read each block of information that contained the critical detail), with the only difference being the inclusion (i.e., the with-context condition) or exclusion (i.e., the without-context condition) of the contextual details.

#### Final memory test

Following a 25 min filled retention interval in which participants completed the Reading Span working memory task [41] and played Tetris, participants were given the final test (which was identical to the initial test). Participants were told to answer the questions based only on their memory for the video. Immediately after each question, participants were asked to rate their confidence in their response from 1 (“I guessed”) to 5 (“I am very sure”). The final test was then followed by a short demographic questionnaire and additional post-experiment questions.

#### Unconstrained cued recall test

Prior to debriefing, participants completed an additional, unconstrained cued recall test (UCR; also known as modified free recall, or MMFR [42]). The UCR test contained the same questions as the final test, but participants were instructed to recall details from both the video and the narrative even if the details contradicted one another. Participants were given 30 s to answer each question and were not asked to specify the source of their recalled details. Their responses were scored in the same way as in the cued recall test, but because the UCR test is designed to elicit multiple responses for the misleading questions, the combined probabilities of *Correct*, *Misinformation*, *Other*, and *No Answer* responses could exceed 1. While the final test only permitted one response, the UCR test did not constrain participants into reporting a single answer for each question, so that it reduced the influence of response competition and removed the requirement of source monitoring and response editing/selection. Because this UCR test was administered after the final recall test, performance on the UCR test was contaminated by the final recall test. This contamination clouds interpretation of results from the UCR test. We therefore included the UCR test for exploratory purposes only. The data for this test are presented in the supporting information (S1 Text) and will not be discussed further.

### Results

#### Initial test

Responses were classified as either *correct*, matching the *misinformation* (spontaneous misinformation recall for the initial test), *no answer* (blank response or “I don’t know”), or *other* response (any response that was incorrect, but did not match the misinformation). See Table 2 for performance on the initial test for all three experiments. The level of correct recall was similar to previous studies (*M* = .57 [12]) and spontaneous misinformation recall was infrequent, as expected (*M* = .03).

**Table 2:** Mean probabilities (and standard deviations) of correct, misinformation, other and no answers on the initial test in experiments 1, 2, and 3.

	Correct	Misinformation	No Answer	Other
**Experiment 1**	.57 (.16)	.03 (.04)	.05 (.06)	.34 (.14)
**Experiment 2**	.59 (.14)	.02 (.04)	.05 (.06)	.34 (.15)
**Experiment 3**	.58 (.16)	.05 (.06)	.07 (.09)	.29 (.12)

#### Final test correct recall

**Fig 1:**
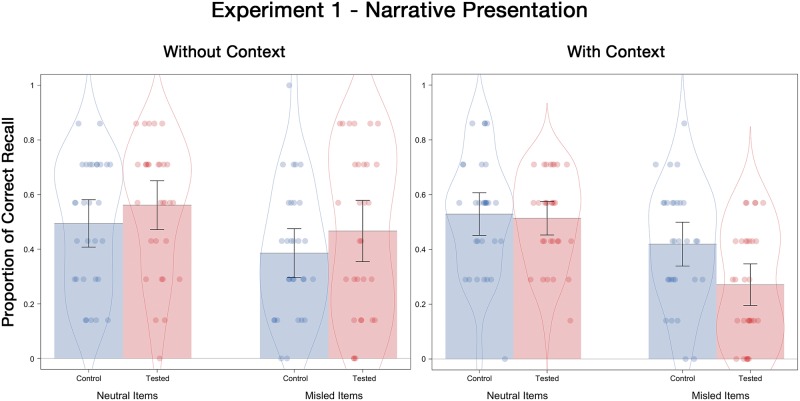
Correct recall probabilities for neutral and misled items as a function of the presence and absence of contextual details on the final test of Experiment 1. Error bars indicate descriptive 95% confidence intervals.

Proportion of correct recall for the final test are presented in Fig 1. Other and No Answer responses for all experiments are included in Table 3. A 2 (tested, control) X 2 (with-context, without-context) X 2 (neutral item, misled item) ANOVA revealed a significant misinformation effect, with participants recalling fewer correct details for misled items (*M* = .39) than for neutral items (*M* = .52), *F*(1, 116) = 30.53, *p* < .001, ƞ^2^_p_ = .21. There was also a significant crossover interaction between initial testing and contextual detail, *F*(1, 116) = 5.49, *p* = .02, ƞ^2^_p_ = .05. Specifically, there was a trend towards a testing effect in the without-context condition (*M* = .51 for tested; *M* = .44 for control), *t*(58) = 1.50, *p* = .14, *d* = .36, but no testing effect in the with-context condition (*M* = .39 for tested; *M* = .47 for control), *t*(58) = 1.84, *p* = .07, *d* = .47. No other interactions or main effects were significant.

**Table 3:** Mean probabilities (and standard deviations) of no answer and other responses on the final test in experiments 1, 2, and 3.

		No Answer				Other Responses			
		**With Context**		**Without Context**		**With Context**		**Without Context**	
		**Neutral**	**Misled**	**Neutral**	**Misled**	**Neutral**	**Misled**	**Neutral**	**Misled**
**Experiment 1**	**Control**	.02 (.05)	.01 (.04)	.06 (.07)	.01 (.04)	.45 (.20)	.21 (.18)	.42 (.20)	.23 (.25)
	**Tested**	.04 (.06)	.01 (.04)	.03 (.06)	.01 (.04)	.43 (.17)	.19 (.15)	.38 (.22)	.17 (.14)
**Experiment 2**	**Control**	.05 (.09)	.04 (.06)	.02 (.05)	.06 (.07)	.40 (.21)	.33 (.19)	.35 (.19)	.20 (.16)
	**Tested**	.03 (.06)	.03 (.06)	.02 (.06)	.01 (.04)	.36 (.15)	.17 (.15)	.36 (.23)	.26 (.18)
		**Coherent**		**Random**		**Coherent**		**Random**	
		**Neutral**	**Misled**	**Neutral**	**Misled**	**Neutral**	**Misled**	**Neutral**	**Misled**
**Experiment 3**	**Control**	.03 (.06)	.03 (.07)	.03 (.07)	.02 (.05)	.38 (.18)	.23 (.15)	.32 (.17)	.29 (.23)
	**Tested**	.05 (.10)	.01 (.04)	.05 (.08)	.02 (.05)	.34 (.19)	.22 (.13)	.35 (.15)	.20 (.18)

#### Final test misinformation recall

**Fig 2:**
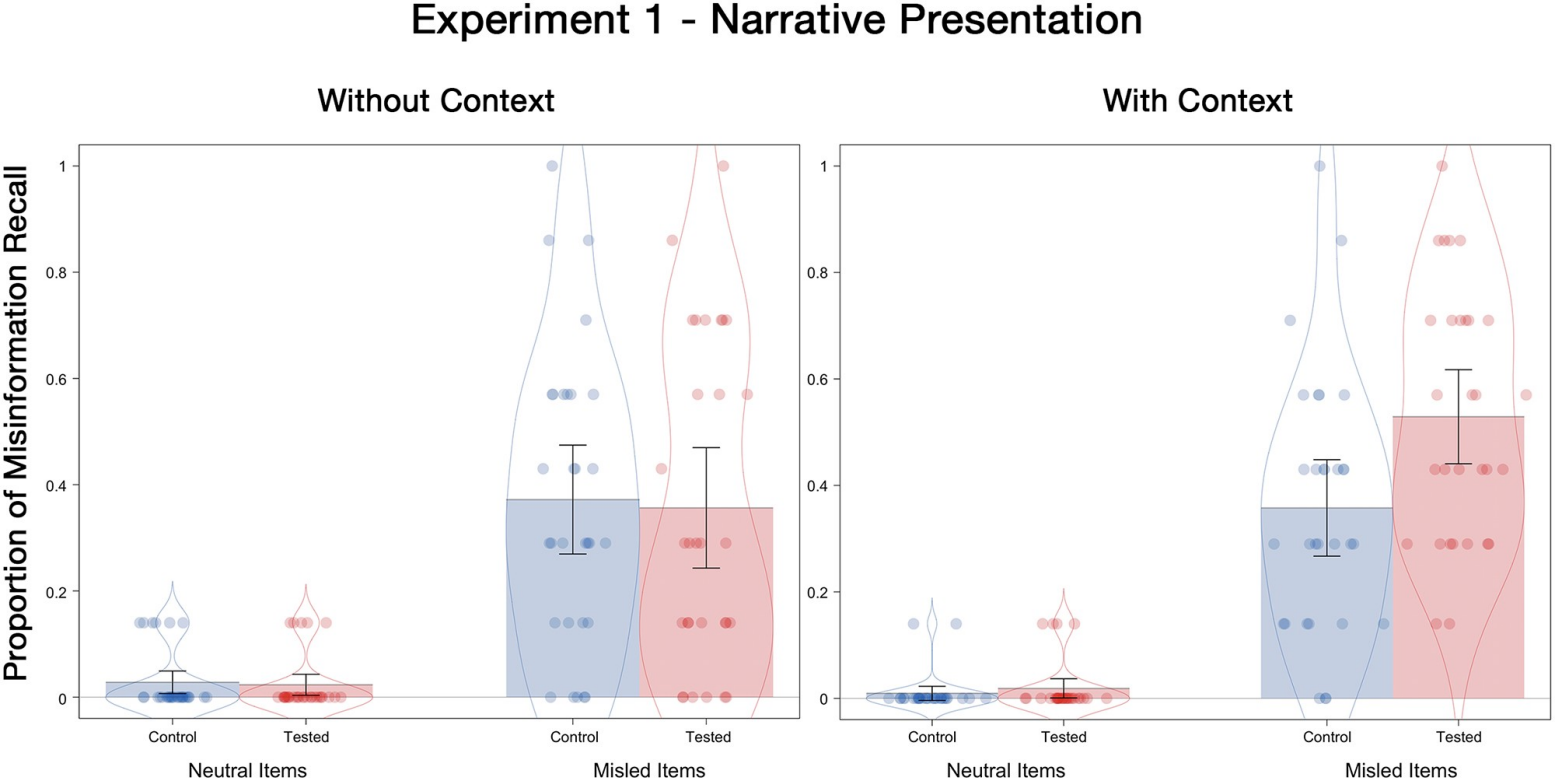
Misinformation recall probabilities for neutral and misled items as a function of the presence and absence of contextual details on the final test of Experiment 1. Error bars indicate descriptive 95% confidence intervals.

Misinformation recall probabilities are displayed in Fig 2. A 2 (tested, control) X 2 (with-context, without-context) X 2 (neutral item, misled item) ANOVA revealed a marginally significant 3-way interaction, *F*(1, 116) = 2.95, *p* = .09, ƞ^2^_p_ = .03. There was also a marginally significant interaction between postevent information and contextual detail, *F*(1, 116) = 3.25, *p* = .07, ƞ^2^_p_ = .03. Misinformation recall was numerically higher for misled items presented with context (*M* = .44) than those presented without context (*M* = .36), *t*(118) = 1.60, *p* = .11, *d* = .30; whereas no difference was found for neutral items (*M* = .01 and *M* = .03 for with- and without-context conditions respectively), *t*(118) = 1.31, *p* = .19, *d* = .24. As expected, there was a significant main effect of postevent information, with misinformation presentation increasing misinformation recall probability from .02 for the neutral items to .40 for the misled items, *F*(1, 116) = 232.40, *p* < .001, ƞ^2^_p_ = .67.

Most important for present purposes was that the interaction between initial testing and contextual detail was significant, *F*(1, 116) = 4.33, *p* = .04, ƞ^2^_p_ = .04. Specifically, there was an RES effect in the with-context condition (*M* = .53 for tested; *M* = .36 for control), *t*(58) = 2.77, *p* = .01, *d* = .71, but not in the without-context condition (*M* = .36 for tested; *M* = .37 for control), *t* < 1, *p* = .83, *d* = .04. Consistent with previous studies (e.g., Chan et al., 2017), embedding misinformation into a full narrative (i.e., the with-context condition) resulted in RES. However, when the contextual details were removed, the RES effect was eliminated, even if the misinformation was presented in a narrative format.

When providing a statement to police, witnesses may be asked to respond only when they are very confident. Therefore, we conducted an additional analysis to examined misinformation recall for only highly confident responses (a confidence rating of 4 or 5—“I am sure” or 5 “I am very sure”). This analysis allowed us to reveal whether the RES effect was driven mainly by low confidence responses, or if it occurs even when participants are very confident in their response. The answer is clear: prior retrieval increased eyewitness suggestibility even amongst these highly confident responses in the with-context condition (*M* = .56 for test and *M* = .38 for control), *t*(57) = 2.17, *p* = .03, *d* = .57. However, no difference was found in the without-context condition (*M* = .34 for test and *M* = .36 for control), *t* < 1, *p* = .77, *d* = .08. Indeed, the misinformation recall rates of the highly confident answers matched the overall pattern closely (see Fig 2).

Conditional analyses were performed to examine misinformation recall on the final test depending on whether participants had successfully retrieved the correct answer or not during the initial test phase. Note that this analysis could only be conducted for the tested participants. As expected, participants were more likely to recall the misinformation on the final test when they had provided an incorrect answer during the initial test for a given item (*M* = .68 with context; *M* = .51 without context), compared to when they were able to provide the correct answer during the initial test (*M* = .43 with context; *M* = .30 without context, *t*(27) = 3.26, *p* = .003, *d* = .62 for the with-context condition and *t*(28) = 2.94, *p* = .01, *d* = .55 for the without context condition). Compared to the control participants (*M* = .36), the tested participants recalled quantitatively more misinformation even when they were able to produce the correct answer initially in the with-context condition. This finding is consistent with previous reports [20]. Intriguingly, however, the same was not true for participants in the without-context condition. Here, when participants produced the correct answer initially (*M* = .30), they were numerically less susceptible to later misinformation than the control participants (*M* = .36). Although these results are informative, they must be interpreted with caution. Conditional analyses like this are subject to item-selection effects because the items that were correctly answered during the initial test were likely easier than the remaining items. Therefore, any comparison made between the initially-correct items from the tested participants and the control participants necessarily involves comparing different pools of stimuli, which makes interpretation difficult. Consequently, we have opted not to conduct inferential statistics on these comparisons.

## Experiment 2

In Experiment 2, we further examined the influence of contextual detail on the suggestibility of tested and control participants. But unlike Experiment 1, misinformation was embedded in questions *for all participants*. In the without-context condition, participants were presented with 14 questions (seven of which included a misleading detail)—a replication of Experiment 2 of LaPaglia and Chan’s [12] study. In the with-context condition, participants saw those same questions, but these questions were interspersed with sentences that reinstated the narrative context of the target event. Specifically, the contextual sentences were identical to those in the narrative presentation conditions in Experiment 1.

### Method

#### Participants

There were 120 participants (60 female; mean age = 19.20, *SD* = 2.22), and 30 participants were included in each between-subjects condition.

#### Materials and procedure

Aside from the fact that all misinformation was presented via questions, Experiment 2 used the same materials and procedure as Experiment 1. Participants in the without-context condition were told that they would be answering some questions about the robbery video that they saw earlier. Participants in the with-context condition were told that they would be reading a narrative recapping the robbery video and they would also be asked some questions about that video. For instance, participants might read a block with a few sentences, followed by a block that contains a question, then read a block with some more sentences, and so on. They were given 30 s to read each block of information and to answer each question.

### Results

#### Initial test

Initial test data were coded in the same manner as the previous experiment (see Table 2 for means). Correct recall probability was similar to that of Experiment 1 (*M* = .59) and spontaneous misinformation recall rarely occurred (*M* = .02).

#### Questions of the misinformation presentation phase

Unlike Experiment 1, misinformation was presented through questions in Experiment 2. In each question, participants were asked to recall a piece of *noncritical detail*, and half of these questions contained a piece of misinformation about the critical detail. Table 4 displays the mean proportion recalled of the noncritical details. A 2 (prior testing) X 2 (contextual detail) X 2 (postevent information) ANOVA showed that none of the main effects or interactions were significant, *F*s < 2.72, *p*s > .10. However, to further examine recall performance during this phase, we conducted separate 2 (prior testing) X 2 (postevent information) ANOVAs for the with- and without-context conditions separately. For participants in the with-context condition, there was a significant benefit of prior testing, *F*(1, 58) = 4.68, *p* = .04, ƞ^2^_p_ = .08, with the tested participants recalling more noncritical details (*M* = .81) than the control participants (*M* = .74), but no effect of postevent information, *F* < 1, *p* = .97, such that participants recalled the same proportion of noncritical details regardless of whether the question included a piece of misinformation or not (*M*s = .77). The two variables did not interact, *F*(1, 58) = 1.96, *p* = .17. In contrast, in the without-context condition, there was no effect of prior testing (*M* = .74 for control and *M* = .75 for tested), *F* < 1, *p* = .81, but there was a marginal effect of postevent information, *F*(1, 58) = 2.92, *p* = .09, ƞ^2^_p_ = .09, with participants recalling slightly more noncritical details when the question did not contain misinformation (*M* = .77) than when it did (*M* = .73). The interaction was not significant. We now report results from the final test.

**Table 4:** Mean probabilities and standard deviations of correct responses on the recall test during the misinformation presentation phase of Experiment 2.

	With Context		Without Context	
	Neutral	Misled	Neutral	Misled
**Control**	.75 (.20)	.72 (.17)	.77 (.17)	.72 (.22)
**Tested**	.80 (.12)	.82 (.10)	.77 (.14)	.73 (.14)

#### Final test correct recall

**Fig 3:**
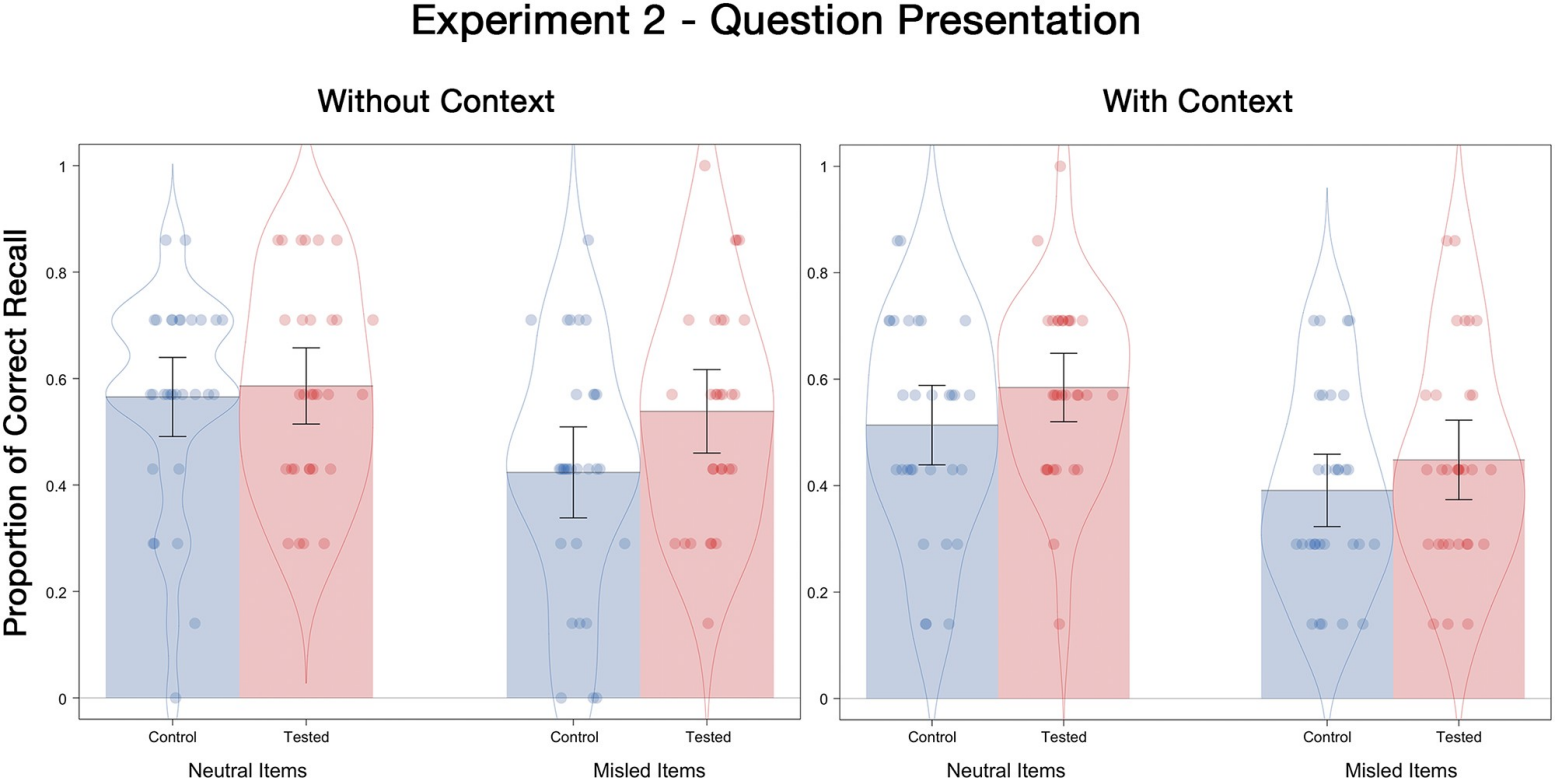
Correct recall probabilities for neutral and misled items as a function of the presence or absence of contextual details on the final test of Experiment 2. Error bars indicate descriptive 95% confidence intervals.

Fig 3 displays the mean correct recall probabilities. A 2 (prior testing) X 2 (contextual detail) X 2 (postevent information) ANOVA showed no significant interactions. There was, however, a significant testing effect (*M* = .53 for tested; *M* = .47 for control), *F*(1, 116) = 5.41, *p* = .02, ƞ^2^_p_ = .05. There was also a significant misinformation main effect, such that presenting misinformation reduced correct recall probability from .56 for neutral items to .45 for misled items, *F*(1, 116) = 24.44, *p* < .001, ƞ^2^_p_ = .17.

#### Final test misinformation recall

**Fig 4:**
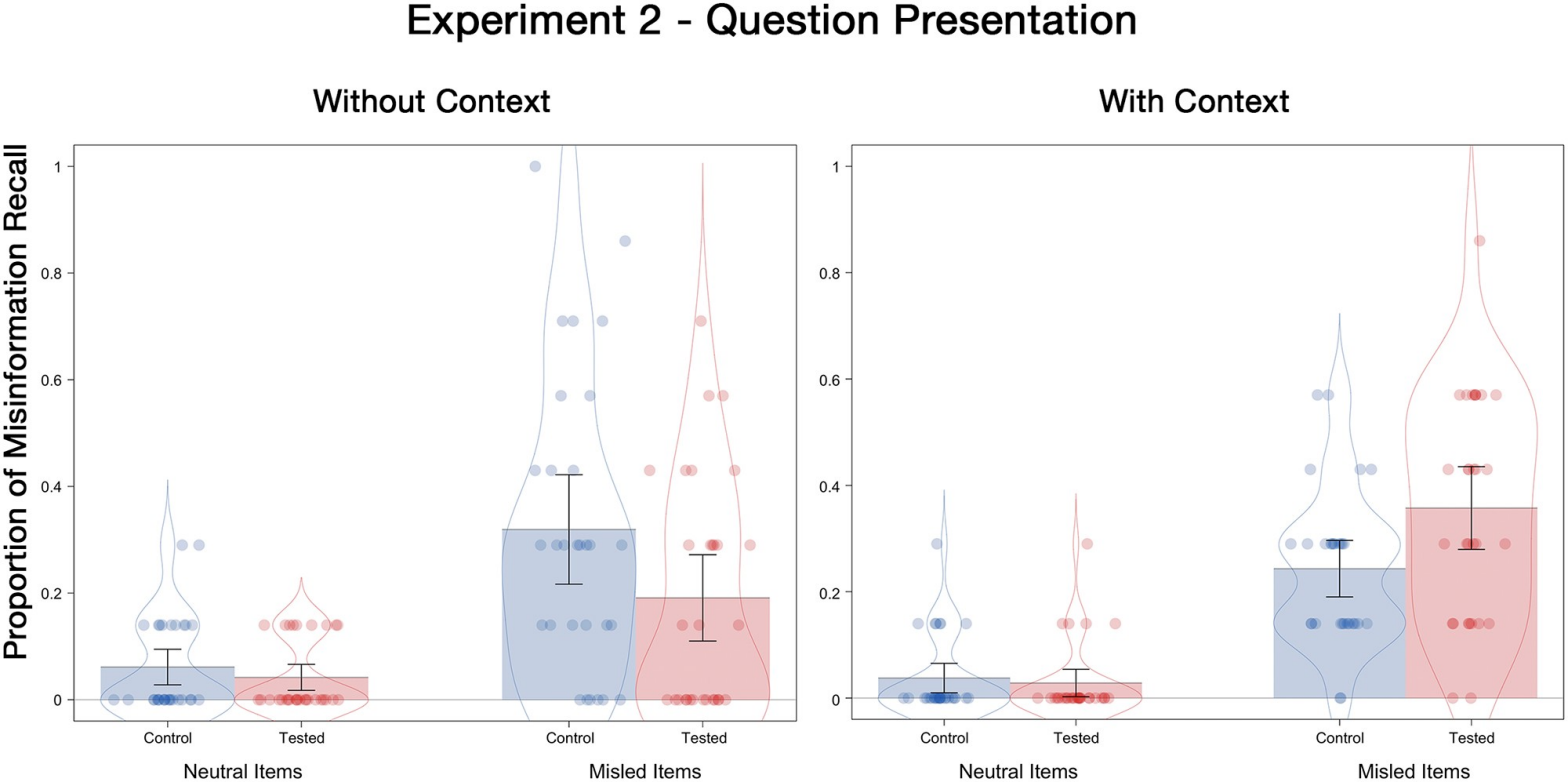
Misinformation recall probabilities for neutral and misled items as a function of the presence or absence of contextual details on the final test of Experiment 2. Error bars indicate descriptive 95% confidence intervals.

Misinformation recall probabilities are displayed in Fig 4. There was a significant 3-way interaction between postevent information, initial testing, and contextual detail, *F*(1, 116) = 6.66, *p* = .01, ƞ^2^_p_ = .05. Not surprisingly, there was a significant misinformation effect, with a higher misinformation recall probability for misled items (*M* = .28) than for neutral items (*M* = .04), *F*(1, 116) = 109.38, *p* < .001, ƞ^2^_p_ = .49. Most importantly, there was an interaction between testing and contextual detail, *F*(1, 116) = 10.97, *p* = .001, ƞ^2^_p_ = .09. Replicating LaPaglia and Chan [20], initial testing reduced suggestibility when the misleading questions were presented without contextual details (*M* = .19 for tested participants and *M* = .32 for control participants), *t*(58) = 2.01, *p* = .05, *d* = .53. However, the opposite occurred when the misleading questions were embedded in a narrative, such that initial testing increased suggestibility when the misleading questions were presented with contextual details (*M* = .36 for tested and *M* = .24 for control; an RES effect), *t*(58) = 2.47, *p* = .02, *d* = .67. Notably, this condition was not included in the aforementioned LaPaglia and Chan study. Indeed, the size of this RES effect was similar to that in Experiment 1 (*d* = .71), which suggests that presenting misinformation via questions or a narrative had little to do with whether RES occurs. Instead, the results of Experiments 1 and 2 made clear that the inclusion or exclusion of contextual details play a key role in RES.

We next examined whether RES occurred when we restricted the analysis to only highly confident responses (i.e., a 4 or 5 on the 5-point confidence scale). Unlike Experiment 1, the RES effect was not significant in the with-context condition (*M* = .37 for test and *M* = .28 for control), *t*(55) = 1.48, *p* = .14, *d* = .39, although the difference remained in the direction of RES. In the without-context condition, retrieval reduced suggestibility amongst highly confident responses (*M* = .16 for tested and *M* = .37 for control), *t*(57) = 2.87, *p* < .01, *d* = .75. Once again, these results mirrored those from the overall analysis.

We now present the results of a conditional analysis that examined misinformation recall on the final test depending on retrieval success during the initial test. Similar to Experiment 1 and as expected, participants were more likely to succumb to misinformation on the final test (*M* = .48 with context; *M* = .31 without context) when they had answered the question incorrectly than when they had answered the question correctly on the initial test (*M* = .23 with context; *M* = .10 without context, *t*(28) = 3.41, *p* = .002, *d* = .63, for the with-context condition and *t*(28) = 3.82, *p* < .001, *d* = .71 for the without context condition). Once again, we caution against over-interpreting these findings due to item-selection artifacts in conditional analyses.

## Experiment 3

Experiments 1 and 2 showed that the elimination of RES when misinformation was presented in questions in LaPaglia and Chan’s [12] study was not due to differences inherent to providing misinformation in sentences vs. questions, but as a result of the misleading questions being presented in an isolated, context-free manner. Experiment 3 was designed to further test the Context Processing Account by manipulating the ease of gist processing without altering the amount of contextual information presented to participants. To accomplish this, all participants received the same amount of contextual information, but we varied gist processing by presenting the narrative blocks either in a coherent (i.e., sequential) order or in a random order. If the elimination of the RES effect in the without-context condition in Experiments 1 and 2 was due to reduced gist processing of the narrative (and thus a greater reliance on the verbatim trace of the original event), then the RES effect should be similarly eliminated in the random context condition.

### Method

#### Participants

Experiment 3 used a 2 (initial test condition: tested vs. control) X 2 (context presentation order: coherent vs. random) X 2 (postevent information: neutral vs. misled) mixed design. Initial test condition and context order were manipulated between subjects. There were 120 participants (63 female; mean age = 19.53, *SD* = 1.68), with 30 in each between-subjects condition.

#### Materials and procedure

Experiment 3 used the same materials and procedure as Experiments 1 and 2 with the exception of the misinformation phase. Critical details were embedded in an experimenter-paced written narrative; however, blocks of information that did not include critical details were either presented in their natural, coherent order or in a random order. Critically, the presentation blocks that contained the critical details were always presented in the same position regardless of condition. Specifically, blocks 1, 4, 6, 8, 10, 12, 15, 16, 17, 21, 22, 24, 25, and 27 contained critical details. There was also no UCR test. After the final cued recall test, participants completed a post-test questionnaire that asked participants to provide retrospective ratings about how they read and thought about the narrative. The results for this post-test questionnaire were largely uninformative and they will not be discussed further.

### Results

#### Initial test

Initial test data were coded in the same manner as the previous experiments (see Table 2 for means). Correct recall probability (*M* = .58) was similar to those in Experiments 1 and 2, and spontaneous misinformation recall was rare (*M* = .05).

#### Final test correct recall

**Fig 5:**
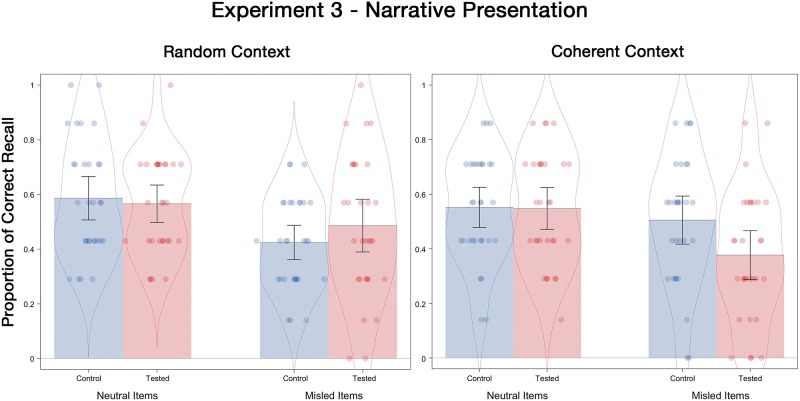
Correct recall probabilities for neutral and misled items as a function of the presentation order of context details on the final test of Experiment 3. Error bars indicate descriptive 95% confidence intervals.

Mean recall probabilities are displayed in Fig 5. A 2 (tested, control) X 2 (context order) X 2 (postevent information) ANOVA showed a significant main effect of misinformation, *F*(1, 116) = 26.94, *p* < .001, ƞ^2^_p_ = .19. That is, presenting misinformation reduced correct recall probability from .56 for neutral items to .45 for misled items. Moreover, there was a significant three-way interaction, *F*(1, 116) = 5.41, *p* = .02, ƞ^2^_p_ = .05. To deconstruct this three-way interaction, we conducted separate 2 (tested, control) X 2 (context order) ANOVAs for neutral items and misled items, respectively. For neutral items, neither main effects nor the interaction were significant, *F*s < 1, *p* > .47. However, for misled items, a crossover interaction emerged, *F*(1, 116) = 5.20, *p* = .02, ƞ^2^_p_ = .04. Specifically, when contextual details were presented in a coherent order, the tested participants recalled fewer correct details (*M* = .38) than the control participants (*M* = .51), *t*(58) = 2.09, *p* = .04, *d* = .54. However, when contextual details were presented in a random order, the tested participants recalled numerically, though not significantly, more correct details (*M* = .49) than the control participants (*M* = .42), *t*(58) = 1.10, *p* = .28, *d* = .28.

#### Final test misinformation recall

**Fig 6:**
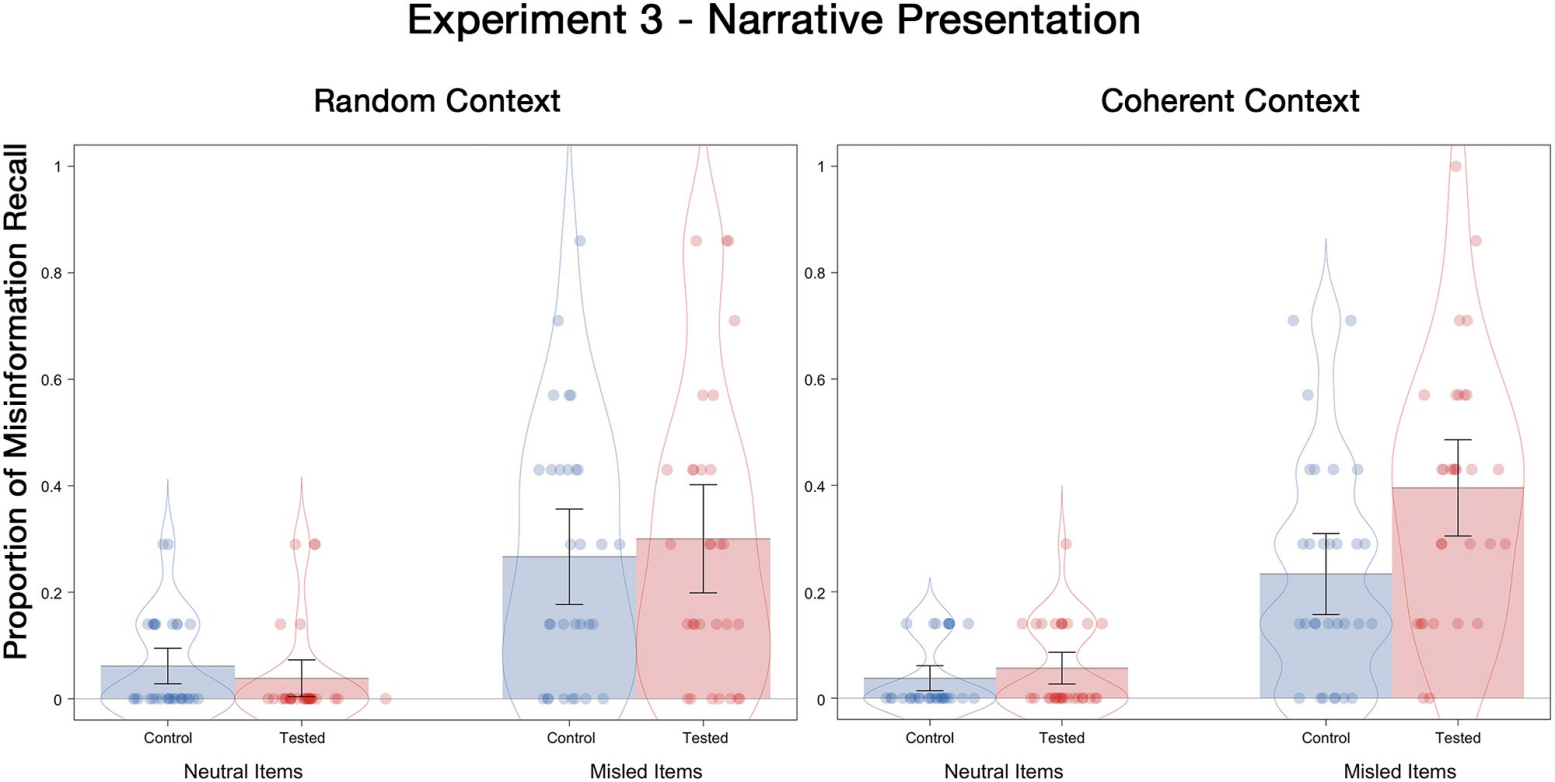
Misinformation recall probabilities for neutral and misled items as a function of the presentation order of context details on the final test of Experiment 3. Error bars indicate descriptive 95% confidence intervals.

Misinformation recall probabilities are displayed in Fig 6. A 2 (tested, control) X 2 (coherent, random) X 2 (neutral, misled) ANOVA showed a significant main effect of misinformation, *F*(1, 116) = 110.90, *p* < .001, ƞ^2^_p_ = .49, with misinformation recall being higher for misled items (*M* = .30) than neutral items (*M* = .05). There was also a main effect of testing, *F*(1, 116) = 4.49, *p* = .04, ƞ^2^_p_ = .04, with the tested participants recalling more misinformation (*M* = .20) than the control participants (*M* = .15), an RES effect overall. The interaction between testing and item type was significant, *F*(1, 116) = 4.40, *p* = .04, ƞ^2^_p_ = .04, such that testing did not affect spontaneous misinformation recall of the neutral items (*M* = .05 for both control and tested), but it produced an RES effect for the misled items, (*M* = .35 for tested and *M* = .25 for control). Lastly, the interaction between testing and context-order was marginally significant, *F*(1, 116) = 3.55, *p* = .06, ƞ^2^_p_ = .03.

To more clearly assess the influence of context order on RES, we conducted separate 2 (tested, control) X 2 (neutral, misled) ANOVAs for the coherent-context and random-context conditions, respectively. In the coherent-context condition, there was a significant interaction between postevent information and initial testing, *F*(1, 58) = 5.99, *p* = .02, ƞ^2^_p_ = .09. Specifically, testing did not affect (spontaneous) misinformation recall probabilities of the neutral items (*M* = .04 for tested and *M* = .06 for control), *t* < 1, *p* = .33, but it produced an RES effect for the misled items (*M* = .40 for tested and *M* = .23 for control participants), *t*(58) = 2.80, *p* = .01, *d* = .77. A different pattern of results emerged when contextual information was presented in a random order—there was no interaction between postevent information and initial testing, *F* < 1, *p* = .45, and most critically, consistent with the prediction emerging from the Context Processing Account, the RES effect was eliminated here (*M* = .30 for tested and *M* = .27 for control), *t* < 1, *p* = .61.

Once again, we examined misinformation recall for only highly confident responses. An RES effect remained for the coherent condition (*M* = .42, for tested and *M* = .28 for control), although this difference was marginal, *t*(58) = 1.77, *p* = .08, *d* = .46. There was no difference between the tested and control groups in the random condition (*M* = .29 for test and *M* = .24 for control), *t* < 1, *p* = .55, *d* = .16.

Next, we examined misinformation recall on the final test depending on retrieval success during the initial test. Similar to the findings in Experiments 1 and 2, participants were more likely to recall the misinformation when they had answered the same question incorrectly previously (*M* = .59 for coherent; *M* = .46 for random) than when they had answered that question correctly earlier (*M* = .29 for coherent; *M* = .22 for random), *t*(29) = 4.67, *p* < .001, *d* = .85 for the coherent condition and *t*(28) = 3.04, *p* = .01, *d* = .57 for the random condition. Once again, we caution against over-interpreting these results because of item selection artifacts.

## General discussion

In many criminal investigations, prosecution of a suspect can rely almost entirely on eyewitness evidence [43]. This is alarming given that eyewitness memory can be contaminated by misinformation, amongst other factors [44]. Witnesses are provided many opportunities to recall details of the crime—i.e., to a 911 operator or a police investigator on scene. Ample evidence has demonstrated that prior recall can have a profound influence on later eyewitness suggestibility, with some studies showing that retrieval can exacerbate the negative impact of misinformation [5] and others showing that retrieval can protect against misinformation [45]. Investigations into initial test format [46,47] and delay [3,18,48] have found that these factors are unlikely to be why researchers sometimes found opposite effects of testing on eyewitness suggestibility. LaPaglia and Chan [12], however, discovered that variations in the method through which misinformation is delivered can have a profound impact on whether or not prior memory retrieval would exacerbate eyewitness suggestibility towards subsequently presented misinformation. Specifically, they found that when misinformation was presented in a narrative, prior retrieval enhanced suggestibility. However, retrieval no longer enhanced suggestibility when misinformation was embedded in misleading questions. In fact, there was a protective effect of testing on memory. The aim of the current study was to clarify why variations in misinformation format can alter the relationship between retrieval and suggestibility.

In this paper, we proposed the Context Processing Account, which suggests that whether prior retrieval would increase eyewitness suggestibility hinges on the type of encoding processes engaged during presentation of the misinformation. If the misinformation presentation method favors gist processing, prior testing should increase suggestibility. In contrast, if the misinformation presentation method favors verbatim processing, prior testing should not increase suggestibility (and may in fact reduce suggestibility). Data from the current three experiments lend support to this account. Specifically, when the misinformation phase reinstated the context of the witnessed event (by including contextual details in a coherent order), and thus promoted gist processing, an RES effect was observed. On the contrary, when the misinformation phase discouraged gist-processing (by removing contextual information or randomizing the order of the contextual information), the RES effect was eliminated. Importantly, we obtained these results regardless of whether the misinformation was embedded in sentences or questions, thereby demonstrating that it is not misinformation presentation format per se (i.e., sentences vs. questions), but the type of encoding processes (i.e., gist vs. verbatim) elicited by the presentation format, that determined whether RES was observed.

Why does a bias toward verbatim processing reduce eyewitness’ susceptibility to misinformation—but only when participants were tested previously? We believe that when verbatim processing is encouraged, participants may opt to more carefully examine the accuracy of the postevent information as it is presented, which would allow the benefits of prior retrieval to be revealed [25,49,50]. The idea that comprehension of the study materials can affect their perceived accuracy is well-documented. For example, McCabe and Castel [51,52] showed that presenting fMRI images with text increases the perceived credibility of the claims made in the text. One explanation of this finding is that the picture increases the perceived ease of processing [53]. Similarly, people find disfluent speakers to be less credible than their fluent counterparts [54,55], and researchers have posited that fluent processing can increase the persuasiveness of narratives [56]. Because prior testing should strengthen the verbatim (and gist) trace of the witnessed event details, the tested participants should be particularly well-equipped to detect discrepancies between the misinformation and their recollection when they are motivated to carefully monitor the postevent information for inaccuracies (such as when gist processing is discouraged). Consistent with this idea, procedures that aimed to increase retrieval monitoring, such as providing a warning about the veracity of the postevent material [8] or forcing participants to identify the source of the retrieved material [57], can eliminate the RES effect.

In sum, we have demonstrated that context can affect how people process misleading information. In the present experiments, we varied processing characteristics of the postevent information by manipulating the inclusion (Experiments 1 and 2) and presentation order (Experiment 3) of the contextual information. From an application perspective, it is conceivable that other variables that affect the reinstatement of the original context, and thus the likelihood of gist processing, might have similar effects on eyewitness suggestibility. For example, as we mentioned previously, people find non-native speakers, who are more difficult to comprehend, to be less credible than native speakers [55,58], even if the two groups utter the same information. Consequently, we speculate that eyewitnesses may be less susceptible to the influence of misinformation when it is presented by a non-native speaker. More broadly, people may find information that is presented in a less fluent manner less believable. For instance, a witness may re-count an event out of chronological order, may describe the event in a disorganized manner, or provide few details. Future research may benefit from a systematic, and broader examination of the impact of context processing on eyewitness suggestibility, juror perceptions, or even the perceived credibility of news, especially those originated from noncredible sources like social media.

### Concluding remarks

Misinformation is becoming increasingly accessible with the proliferation of online media outlets [59]. With a reported 62% of U.S. adults getting news from social media (which has inadequate fact-checking mechanisms) and the rising popularity of fake news on the internet, empirical studies on the misinformation effect are timelier than ever [60]. In an attempt to reduce the damaging effects of misinformation on memory, researchers have examined the influence of testing on suggestibility and have found mixed results [5,61]. LaPaglia and Chan [12] discovered that the misinformation format alters the relationship between testing and suggestibility.

The objective of the current study was to examine under what conditions retrieval enhances or reduces suggestibility, which is key to leveraging the beneficial effects of retrieval to enhance the reliability of eyewitness accounts. According to the Department of Justice’s eyewitness interview guidelines, investigators should attempt to “conduct [an] interview as soon as the witness is physically and emotionally capable” [62]. However, the finding of retrieval-enhanced suggestibility (RES) shows that an early interview may, under some circumstances, increase the witness’ susceptibility to subsequent misinformation. To be clear, we believe that obtaining an early interview with an eyewitness is sound advice, because misinformation exposure is often out of the control of the criminal justice system (e.g., a witness may talk about the case with another witness, which can lead to contamination of each other’s accounts). However, given the potential ramifications of RES in criminal trials, achieving a better understanding of the conditions under which RES is more and less likely to occur represents the first step to devising strategies that can maximize the benefits of an early interview and avoid its potential pitfalls.

## Supporting information

S1 Appendix Misinformation narrative. (DOCX)

S1 Text Results from the unconstrained cued recall test for experiments 1 and 2. (DOCX)
