# Supplementary material for: Telling a good story: The effects of memory retrieval and context processing on eyewitness suggestibility
Source: PLoS One. 2019 Feb 21;14(2):e0212592. doi: 10.1371/journal.pone.0212592 (PMC6383884; doi:10.1371/journal.pone.0212592)
Supplement: S1 Appendix — (DOCX) [file pone.0212592.s001.docx]

**S1 Appendix**

**Misinformation narrative**

**A single counterbalance of the blocks of information presented in the narrative phase of Experiment 1, 2, and 3. Each solid line indicates a new screen. The misinformation is italicized and the neutral item is underlined. Asterisks (**) signify that the block contains a critical detail. In Experiment 2, the critical detail blocks were replaced with the misleading questions. In Experiment 3, the blocks without asterisks were randomized while the critical blocks remained in the same position.**

**In the first scene in the bank, the manager, Ruth Skellar, is meeting with employees discussing the bank’s ranking in the area.

She explains that the bank’s performance has vastly improved over the last 12 months and congratulates Wendy Trailer for having the top sales in their division.

During the meeting, an employee named Paul, walks in late and begins to apologize. Once Ruth dismisses everyone, she asks to have a word with Paul. He attempts to apologize again but Ruth cuts him off asking him to straighten out his priorities and Paul leaves.

**Ruth asks that someone at the front desk call the security company because *the alarm had mistakenly gone off earlier in the day*.

She proceeds into her office when the door is shut behind her. A robber dressed in all black and a ski mask points a gun at her, hands her two bags, and asks for $500,000 in cash. He asks Ruth to repeat his request then rushes her out of the room, gun still pointed at her.

**Ruth is greeted by an employee who requests to go to her office. She says no, attempting to conceal her fear, and informs the employee of the burglary in progress.

This employee sees a robber in the window and informs Wendy that there is a robbery. Wendy hits the alarm system and everyone in the bank rushes to the door. Just as Ruth is about to leave, the robber grabs her and points the gun at her head, telling her to calm everyone down. The police rush to the bank.

**Sergeant Gregory Parker and two other cops arrive on the scene in a *sedan*.

They discuss what information they have on the guy, but someone disabled video and audio that morning.

**The police assume that this robber is a career bank robber who robbed six banks previously.

The cops discuss ways to enter the bank without the robber seeing them. Meanwhile, inside the bank a phone rings. The robber takes notice and answers, greeted by the police.

**The robber demands that the police get him his money and a car in *one hour*.

He then hands the phone to Ruth and points a gun at her head. She informs the police that the robber will kill them all if they don’t follow his requests.

The cops call the robber back and try to get inside his head, gathering as much information about the robber as possible. After the phone call, Ruth attempts to reason with the robber, informing him that some of them need to use the restroom. He points a gun at her head and she sits down quietly.

**The police make their way through the tunnels and blow up a hole in the wall to the bank, masking it with the alarm sound.

**Inside the bank, Ruth says that a woman with asthma needs her medication. This angers the robber so he fires *two warning shots* into the ceiling.

**The police enter the building. The robber grasps Ruth and threatens to shoot her.

The cops all leave with the other hostages and the robber has Ruth lock the door. He then grabs her and heads up the stairs near her office. He throws her against the wall and his mask comes off, revealing his identity. Ruth recognizes him as a man named George.

The scene cuts to the police who have reconnected the video in the bank, but not the audio. They see a video clip of the robber with his face revealed and Ruth in the hallway near her office. They call in the bank security guard who identifies the man as George Orstin, who was the head of security at the bank. Two months ago Ruth fired him.

The police begin to go over his file and all of the information they have on him. A doctor offers advice of how to deal with a disgruntled employee. The police discuss the situation with Wendy and Paul. They inform the police that Ruth came in and saved the bank a year ago, but has let go of numerous employees. They sympathize with George’s feelings since they don’t care for Ruth either.

**Ruth and George are still in the *beige* hallway. Ruth is sitting on the left side.

**Sgt. Parker calls George using a wireless phone and tries to calm him down by empathizing with him.

George is angry so he demands that he gets what he wants in half an hour or he will kill Ruth. He demands that the money be transferred into an account. George is angry with Ruth and wants to know how she could take a 20 year employee and kick him out of the company. Ruth says it was standard procedure, nothing personal. The police try and relate George to Ruth but he hangs up.

**Outside the bank, the security guard says that all the robber said when he was fired was that he would now have more time to spend with his wife, *Sarah*.

**Meanwhile, the police learn more about the account that began with 0-6 that George had given to Sgt. Parker.

The cops continue their banter with George. Ruth cuts in and tells George she hates firing people and hates that she knows everyone dislikes her. She continues in saying that she worked hard to build the bank up so everyone could keep their jobs.

**If it wasn’t for Ruth, *55* people would have lost their jobs, yet she only fired 7 people.

She apologizes to George and he hangs up on the police. He says, “No more talking!” obviously agitated that he is feeling bad for Ruth.
