# Supplementary material for: Telling a good story: The effects of memory retrieval and context processing on eyewitness suggestibility
Source: PLoS One. 2019 Feb 21;14(2):e0212592. doi: 10.1371/journal.pone.0212592 (PMC6383884; doi:10.1371/journal.pone.0212592)
Supplement: S1 Text — (DOCX) [file pone.0212592.s002.docx]

**S1 Text**

Results from the Unconstrained Cued Recall Test for Experiments 1 and 2 are provided below.

**Experiment 1**

**Correct recall**. A 2 (tested, control) X 2 (contextual detail) X 2 (postevent information) ANOVA resulted in a significant interaction between postevent information and contextual detail, *F*(1, 116) = 6.49, *p* = .01, ƞ^2^_p_ = .05. Misinformation presentation reduced correct recall in the with-context condition (*M* = .45, *SD* = .24 for misled items; *M* = .57, *SD* = .21 for neutral items), *t*(59) = 3.73, *p* < .001, *d* = .53, but not in the without-context condition, *t* < 1, *p* = .32.

**Misinformation recall.** The interaction between postevent information and contextual detail was significant, *F*(1, 116) = 8.77, *p* = .004, ƞ^2^_p_ = .07. There was an RES effect in the with-context condition (*M* = .57, *SD* = .28 for control and *M* = .70, *SD* = .22 for tested), *t*(58) = 2.04, *p* = .05, *d* = .52, but not in the without-context condition, *t* < 1, *p* = .76. There was also a significant main effect of misinformation (*M* = .64, *SD* = .26 for misled items and *M* = .05, *SD* = .08 for neutral items), *F*(1, 116) = 377.17, *p* < .001, ƞ^2^_p_ = .77, and a significant main effect of contextual detail, with participants recalling more misinformation when it had been presented in a full narrative (*M* = .63, *SD* = .26) than when it was presented without context (*M* = .51, *SD* = .28), *F*(1, 116) = 4.55, *p* = .04. ƞ^2^_p_ = .04.

**Experiment 2**

**Correct recall.** A 2 (tested, control) X 2 (contextual detail) X 2 (postevent information) ANOVA resulted in no significant interactions. There was, however, a significant main effect of postevent information, with fewer correct details recalled for misled items (*M* = .51) than for neutral items (*M* = .56), *F*(1, 116) = 6.02, *p* = .02, ƞ^2^_p_ = .05. There was also a significant testing effect (*M* = .57 for tested and *M* = .50 for control), *F*(1, 116) = 6.57, *p* = .01, ƞ^2^_p_ = .05.

**Misinformation recall.** There was a significant misinformation effect (*M* = .36 for misled items and *M* = .08 for neutral items), *F*(1, 116) = 122.13, *p* < .001, ƞ^2^_p_ = .51. Moreover, the interaction between postevent information and initial testing was marginally significant, *F*(1, 116) = 2.93, *p* = .08, ƞ^2^_p_ = .03. Planned comparisons showed an RES effect in the with-context questions condition (*M* = .46 for tested and *M* = .33 for control), *t*(58) = 2.50, *p* = .02, *d* =.65. However, there was no difference in the without-context condition (*M* = .33 for both the control and tested conditions), *t* < 1, *p* = 1.
